# A latent measure explains substantial variance in white matter microstructure across the newborn human brain

**DOI:** 10.1007/s00429-017-1455-6

**Published:** 2017-06-06

**Authors:** Emma J. Telford, Simon R. Cox, Sue Fletcher-Watson, Devasuda Anblagan, Sarah Sparrow, Rozalia Pataky, Alan Quigley, Scott I. Semple, Mark E. Bastin, James P. Boardman

**Affiliations:** 1 MRC Centre for Reproductive Health, University of Edinburgh, 47 Little France Crescent, Edinburgh, EH16 4TJ UK; 2 Department of Psychology, Centre for Cognitive Ageing and Cognitive Epidemiology, University of Edinburgh, Edinburgh, EH8 9JZ UK; 3 Scottish Imaging Network, A Platform for Scientific Excellence (SINAPSE) Collaboration, Edinburgh, UK; 4 Centre for Clinical Brain Sciences, University of Edinburgh, Chancellor’s Building, 49 Little France Crescent, Edinburgh, EH16 4SB UK; 5 Department of Radiology, Royal Hospital for Sick Children, 9 Sciennes Road, Edinburgh, EH9 1LF UK; 6 University/BHF Centre for Cardiovascular Science, Queen’s Medical Research Institute, University of Edinburgh, Edinburgh, EH16 4TJ UK; 7 Clinical Research Imaging Centre, Queen’s Medical Research Institute, University of Edinburgh, Edinburgh, UK

**Keywords:** Neonate, Brain, Magnetic resonance image, Tractography, Preterm

## Abstract

A latent measure of white matter microstructure (*g*_WM_) provides a neural basis for information processing speed and intelligence in adults, but the temporal emergence of *g*_WM_ during human development is unknown. We provide evidence that substantial variance in white matter microstructure is shared across a range of major tracts in the newborn brain. Based on diffusion MRI scans from 145 neonates [gestational age (GA) at birth range 23^+2^–41^+5^ weeks], the microstructural properties of eight major white matter tracts were calculated using probabilistic neighborhood tractography. Principal component analyses (PCAs) were carried out on the correlations between the eight tracts, separately for four tract-averaged water diffusion parameters: fractional anisotropy, and mean, radial and axial diffusivities. For all four parameters, PCAs revealed a single latent variable that explained around half of the variance across all eight tracts, and all tracts showed positive loadings. We considered the impact of early environment on general microstructural properties, by comparing term-born infants with preterm infants at term equivalent age. We found significant associations between GA at birth and the latent measure for each water diffusion measure; this effect was most apparent in projection and commissural fibers. These data show that a latent measure of white matter microstructure is present in very early life, well before myelination is widespread. Early exposure to extra-uterine life is associated with altered general properties of white matter microstructure, which could explain the high prevalence of cognitive impairment experienced by children born preterm.

**Electronic supplementary material:**

The online version of this article (doi:10.1007/s00429-017-1455-6) contains supplementary material, which is available to authorized users.

## Introduction

White matter tracts connecting cortical networks are fundamental substrates of higher cognitive function in humans. ‘Disconnection’ of networks, which can be inferred from the microstructural properties of tracts, characterizes a number of diseases and contributes to functional impairment through reduced information transfer efficiency (Bartzokis et al. 2004; Penke et al. 2010; Ritchie et al. 2015b; Ball et al. 2015; Uddin et al. 2013; Liston et al. 2011). Tract connectivity has been widely investigated in vivo using diffusion magnetic resonance imaging (dMRI) which is a non-invasive method that provides voxel-wise measures of water molecule diffusion. Since the molecular motion of water in the brain is influenced by biological factors including macromolecules, axonal diameter, membrane thickness and myelination, dMRI enables inference about underlying tract microstructure (LeBihan et al. 1986; Basser and Pierpaoli 1996).

In adulthood, microstructural properties of white matter are shared among major tracts (for example, an adult individual with high fractional anisotropy (FA) in one tract is likely to have high FA in all other tracts in the brain). This property allows for the derivation of a general factor, *g*_FA_, of white matter microstructure (Penke et al. 2010; Cox et al. 2016). The general factor explains almost half of variance in microstructure across major tracts, and latent variable statistical analyses show that *g*_FA_ is predictive of information processing speed and intelligence (Penke et al. 2010, 2012; Ritchie et al. 2015b). The temporal emergence of *g*_FA_ and other general factors of water diffusion biomarkers during human development is unknown and, therefore, its role in the ontogeny of human cognition has not been investigated.

Probabilistic neighbourhood tractography (PNT) is an automatic segmentation technique based on single seed point tractography, that can identify the same fasciculus-of-interest across a group of subjects by modelling how individual tracts compare with a predefined reference tract in terms of length and shape (Clayden et al. 2011). The method has been optimized for use with neonatal dMRI data, which enables tract-averaged measurements of mean ‹*D*›, axial (*λ*_ax_) and radial (*λ*_rad_) diffusivities, and FA, for the major white matter fasciculi during early brain development (corticospinal tracts, genu and splenium of corpus callosum, cingulum cingulate gyri, inferior longitudinal fasciculi) (Anblagan et al. 2015).

Early exposure to extra-uterine life by preterm birth is a leading cause of cognitive impairment in childhood and is strongly associated with a ‘disconnectivity’ phenotype that combines diffuse white matter injury and volume reduction of connected structures (Inder et al. 1999; Boardman et al. 2006; Volpe 2009; Ball et al. 2012). Altered development of thalamocortical networks in association with preterm birth is reported (Boardman et al. 2006; Ball et al. 2013, 2015; Toulmin et al. 2015), but structural and functional connectivity analyses in the newborn period and studies of adults born preterm suggests that network disruption is more widely distributed (Pandit et al. 2014; van den Heuvel et al. 2015; Smyser et al. 2016; Froudist-Walsh et al. 2015; Cole et al. 2015). This raises the hypothesis that disconnectivity in the context of preterm birth is a global rather than localized process.

Preterm birth is associated with an atypical social cognitive profile (Ritchie et al. 2015a). Early social cognition is also extremely tractable to measurement in infancy via measurement of gaze behaviour to social and non-social visual content. For example, visual attention is given to faces very soon after birth, with specific preference to the eye region, while at around 6–9 months a preference for looking at faces in multiple object arrays or animated scenes develops (Johnson et al. 1991; Farroni et al. 2002; Gliga et al. 2009). In addition, eye-movement recordings in response to social stimuli have been used to identify early behavioral trajectories associated with autism (Jones and Klin 2013), to link emergent social cognition with white matter microstructure in specific tracts (Elison et al. 2013), and to distinguish between the social cognitive profiles of infants born preterm and at term (Telford et al. 2016).

We tested the following hypotheses: first, a latent measure of general white matter microstructure (*g*_WM_) is present in the newborn; second, preterm birth is associated with global disconnectivity; and third, that *g* measured in the newborn period is associated with emergent social cognitive function in infancy.

## Materials and methods

### Participants

145 neonates (gestational age at birth range 23^+2^–41^+5^ weeks) were recruited from the Royal Infirmary of Edinburgh between February 2013 and August 2015 to a longitudinal study of the effect of preterm birth on brain structure and long-term outcome. Infants had diffusion MRI (dMRI) at term equivalent age (mean GA 40^+5^ weeks, range 37^+5^–47^+1^) and 83 took part in eye-tracking assessment 6–12 months later (median age 7.9 months, IQR 6.8–8.8).

To study the effect of preterm birth on white matter microstructure the group was divided into those with GA at birth <35 weeks (*n* = 109), and healthy controls recruited from postnatal wards with GA 37–42 weeks (*n* = 36). Exclusion criteria included major congenital malformations, chromosomal abnormalities, congenital infection, overt parenchymal lesions (cystic periventricular leukomalacia, hemorrhagic parenchymal infarction) or post-hemorrhagic ventricular dilatation. Demographic information is shown in Table 1. Ethical approval was obtained from the National Research Ethics Service (South East Scotland Research Ethics Committee 02) and informed consent was obtained from the person with parental responsibility for all individual participants included in the study.

**Table 1:** Clinical and demographic features of the whole group, and the preterm and term controls

	Whole sample (*n* = 145)	Preterm (*n* = 109)	Term (*n* = 36)
Mean PMA at birth/weeks (range)	31^+5^ (23^+2^–41^+5^)	29^+0^ (23^+2^–34^+6^)	39^+6^ (37^+2^–41^+5^)
Mean PMA at scan/weeks (range)	40^+5^ (37^+5^–47^+1^)	40^+0^ (37^+5^–44^+0^)	42^+1^ (39^+0^–47^+1^)
Mean birth weight/kg (sd)	1.72 (1.05)	1.14 (0.24)	3.46 (0.45)
Median age at eye-tracking assessment/months (IQR)	7.9 (6.8–8.8)	7.7 (6.7–8.4)	8.4 (7.7–9.1)
Gender (female:male)	69:76	54:55	15:21

Of the preterm group: 7% had intra-uterine growth restriction (IUGR) defined as a birth weight under the third centile for gender and gestation and 31% had bronchopulmonary dysplasia defined as need for supplementary oxygen at 36 weeks’ PMA. PMA; postmenstrual age.

### Image acquisition

A Siemens MAGNETOM Verio 3 T MRI clinical scanner (Siemens Healthcare Erlangen, Germany) and 12-channel phased-array head coil were used to acquire: T1-weighted MPRAGE (TR = 1650 ms, TE = 2.43 ms, inversion time = 160 ms, flip angle = 9°, voxel size = 1 × 1 × 1 mm^3^, and acquisition time = 7 min 49 s); T2-weighted SPACE (TR = 3800 ms, TE = 194 ms, flip angle = 120°, voxel size = 0.9 × 0.9 × 0.9 mm^3^, acquisition time = 4 min 32 s); dMRI using a protocol consisting of 11 T2- and 64 diffusion-weighted (*b* = 750 s/mm^2^) single-shot spin-echo echo planar imaging (EPI) volumes acquired with 2 mm isotropic voxels (TE = 106 ms and TR = 7300 ms). Infants were scanned without sedation in natural sleep using the feed-and-wrap technique. Physiological stability was monitored using procedures described by Merchant et al. (2009). Ear protection was provided for each infant (MiniMuffs, Natus Medical Inc., San Carlos, CA).

### Image analysis

For all four imaging biomarkers (FA, MD, *λ*_ax_ and *λ*_rad_), tract-averaged values were derived from eight major fasciculi segmented using probabilistic neighbourhood tractography (PNT) optimized for neonatal dMRI data (Bastin et al. 2010; Clayden et al. 2007; Anblagan et al. 2015). In summary after conversion from DICOM to NIfTI-1 format, the dMRI data were preprocessed using FSL tools (http://www.fmrib.ox.ac.uk/fsl) to extract the brain and eliminate bulk patient motion and eddy current-induced artifacts by registering the diffusion-weighted to the first T2-weighted EPI volume of each subject. Using DTIFIT, MD and FA volumes were generated for each subject. From the underlying white matter connectivity data, eight major white matter fasciculi thought to be involved in cognitive functioning were segmented: genu and splenium of corpus callosum, left and right cingulum cingulate gyrus (CCG), left and right corticospinal tracts (CST), and left and right inferior longitudinal fasciculi (ILF). As described in detail in the study by Anblagan et al. (2015), this involved using reference tracts created from a group of 20 term controls.

### Cognitive testing

Infant social cognitive ability was assessed by tracking eye gaze in response to visual social stimuli using methods described by Telford et al. (2016). Infants were positioned on the care-giver’s lap 50–60 cm from a display monitor used to present social stimuli of three levels of complexity: a static face, a face in an array of non-social objects, and a pair of naturalistic scenes with and without social content. Proportional looking time to social content relative to the overall stimulus was recorded using a Tobii© ×60 eye-tracker, and Tobii Studio© (version 3.1.0) software was used for analysis. Because social preference scores that represent the distribution of fixation to social versus general image content are highly correlated across tasks, we combined social preference score from each task into a composite score per participant (Gillespie-Smith et al. 2016).

### Statistical analysis

One principal component analysis (PCA) was conducted for each of the four water diffusion parameters (MD, FA, *λ*_ax_ and *λ*_rad_) across the eight tracts, to quantify the proportion of shared variance between them (i.e. to determine whether a clear single-component solution was present, in line with previous reports in adults). That is, four separate data matrices (one for each DTI parameter) were separately analysed, each with dimensions *n* × *m* where *n* = 145 (number of subjects) and *m* = 8 (tract-averaged values for eight tracts). Thus, each PCA included data from all participants, and all available tracts were included; where tract data were missing (median 3.5% of tracts, IQR 3.5–13.25), the mean FA, MD, *λ*_ax_ and *λ*_rad_ of the group was used to impute values for the missing tract. Next, we examined the effect of preterm birth on differences in these four general water diffusion measures. Initially, we used a dichotomous group design, comparing differences between preterm infants’ and controls’ white matter microstructure (corrected for age at MRI scan) using Welch’s unpaired *t* tests. We then applied linear regression across the entire group to quantify the dose effect of birth term on each measure of microstructure, including PMA at MRI scan and sex as covariates in the model. To compare tract loadings (correlations between the manifest variable and extracted component score) for each tract between preterm infants and controls, we used Fisher’s test of correlation magnitude differences among independent groups (cocor.indep.groups in the cocor package in R) (Diedenhofen and Musch 2015). Finally, we examined associations between white matter microstructure and social cognitive performance using linear regression. The MRI and cognitive variables were corrected for differences in age at their respective data collection points prior to insertion into the model, where gender and group status (preterm/control) were covariates. Statistical analyses were carried out using SPSS v 21.0 (Chicago, IL), and R (https://www.r-project.org) version 3.2.2 (Fire Safety).

## Results

### General component of white matter microstructure

**Fig. 1:**
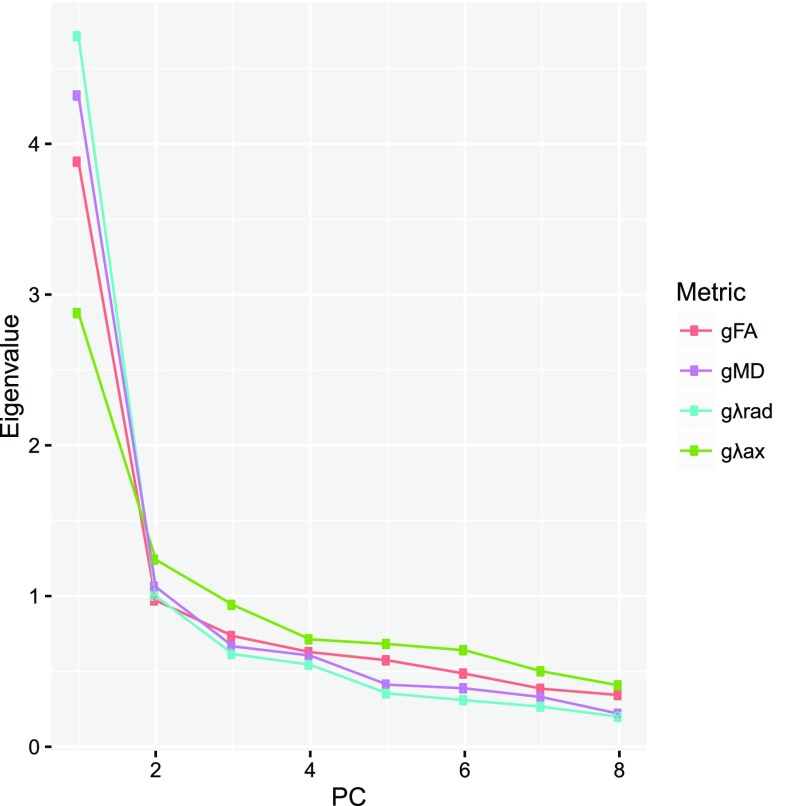
Scree plot from principal component analyses for fractional anisotropy (FA), mean diffusivity (MD), axial diffusivity (*λ*_ax_), and radial diffusivity (*λ*_rad_) of the eight white matter tracts

**Fig. 2:**
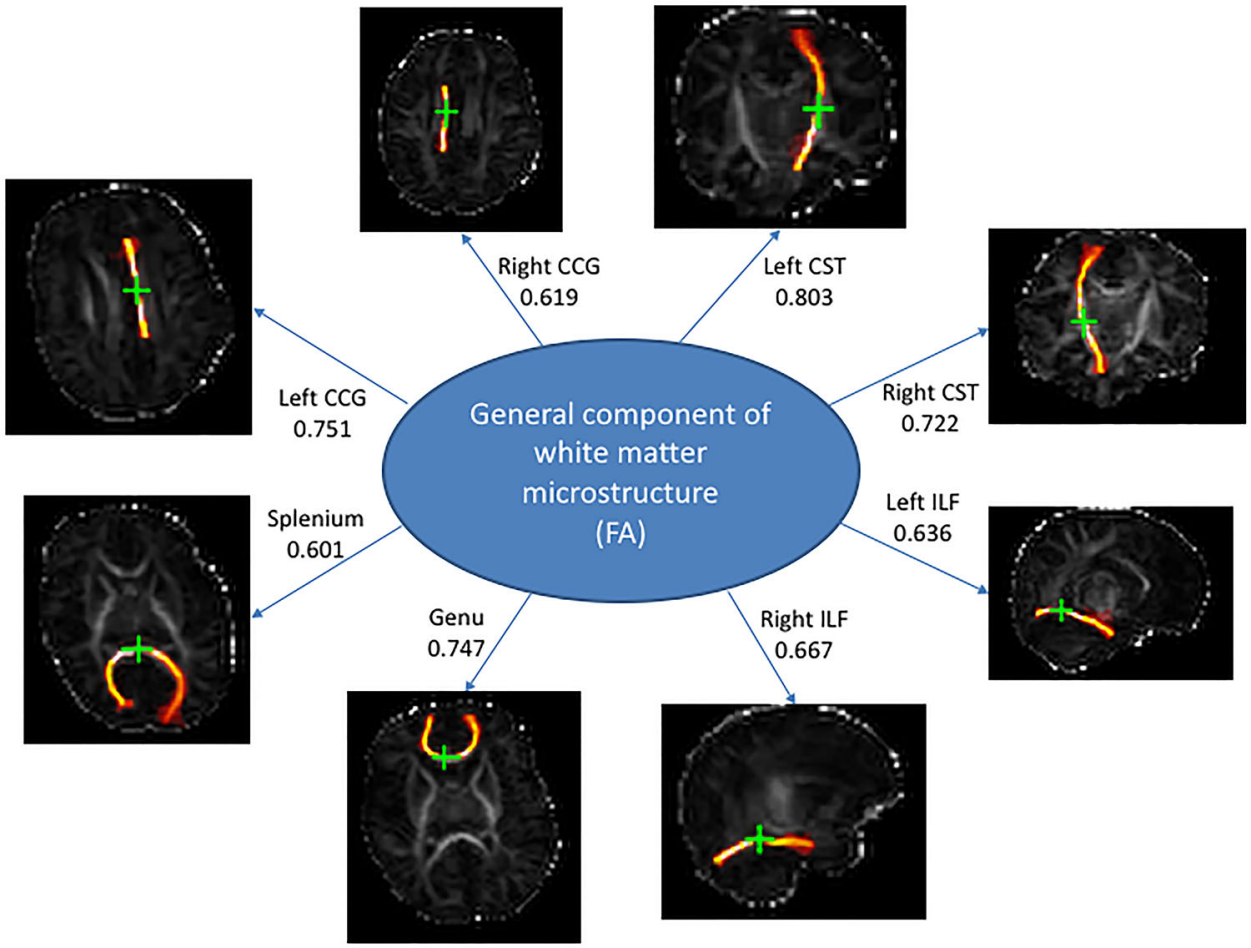
Brain images show tract segmentations obtained from one representative participant. Seed points are marked with a *green cross*. The statistics are loadings of the average FA values of each tract on the latent measure of white matter microstructure

We ran separate PCAs for each measure of white matter microstructure on all eight tracts (Fig. 1). In each case there was a clear one component solution, denoted by its large eigenvalue, and the much lower and linearly decreasing eigenvalues of the remaining components. We extracted this first component, without rotation, which explained 49% (FA), 54% (MD), 59% (*λ*_rad_), and 36% (*λ*_ax_) of the variance (all loadings range between 0.409 and 0.870; Fig. 2; Table 2). Thus, there is a clear tendency for white matter microstructural properties found in one part of the newborn brain to be common across all white matter tracts, and the extracted water diffusion parameter values for each participant, therefore, reflect the level of white matter microstructure common across all tracts in that brain.

**Table 2:** Tract loadings, explained variance, and mean absolute magnitude (Pearson’s *r*) of correlations across all tracts for the first unrotated principal component for the four water diffusion measures

Tract	FA	MD	*λ*_rad_	*λ*_ax_
Genu	0.747	0.841	0.870	0.608
Splenium	0.601	0.737	0.750	0.672
L CCG	0.751	0.738	0.807	0.564
R CCG	0.619	0.783	0.808	0.639
L CST	0.803	0.739	0.812	0.409
R CST	0.722	0.768	0.784	0.652
L ILF	0.636	0.726	0.741	0.669
R ILF	0.667	0.500	0.516	0.538
Explained variance	0.485	0.540	0.589	0.360
Mean between-tract *r*	0.407	0.466	0.521	0.262

*CCG* cingulum cingulate gyri, *CST* corticospinal tract, *ILF* inferior longitudinal fasciculus

## The effect of preterm birth on the general measure of white matter microstructure

**Fig. 3:**
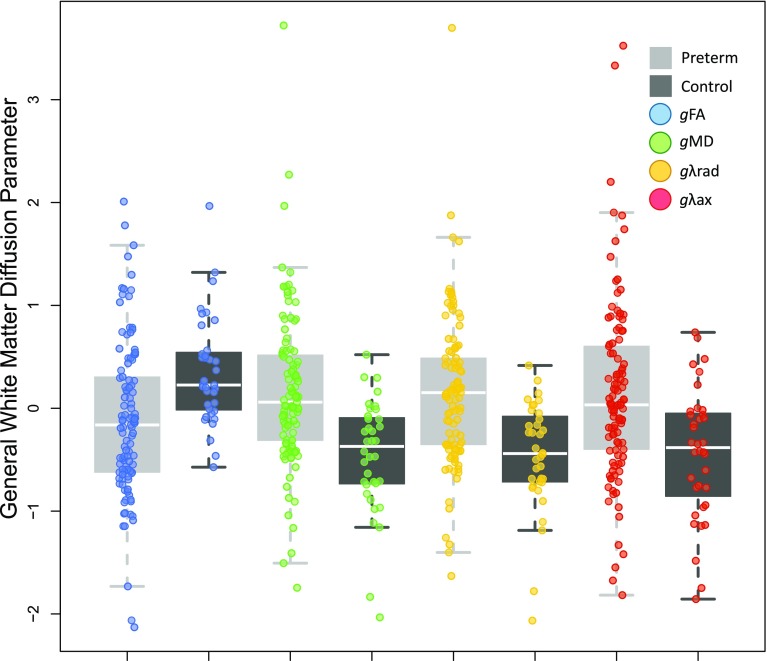
Significant group differences between preterm and controls across all four general water diffusion indices

There were significant differences in *g* for each of the four white matter water diffusion parameters between preterm and control groups: *g*_FA_ (*t* = −4.1367, *p* = 8.139e−05); *g*_MD_ (*t* = 5.2773, *p* = 1.062e−06); *gλ*_rad_ (*t* = 5.4887, *p* = 4.322e−07); *gλ*_ax_ (*t* = 4.2527, *p* = 5.529e−05), Fig. 3.

**Fig. 4:**
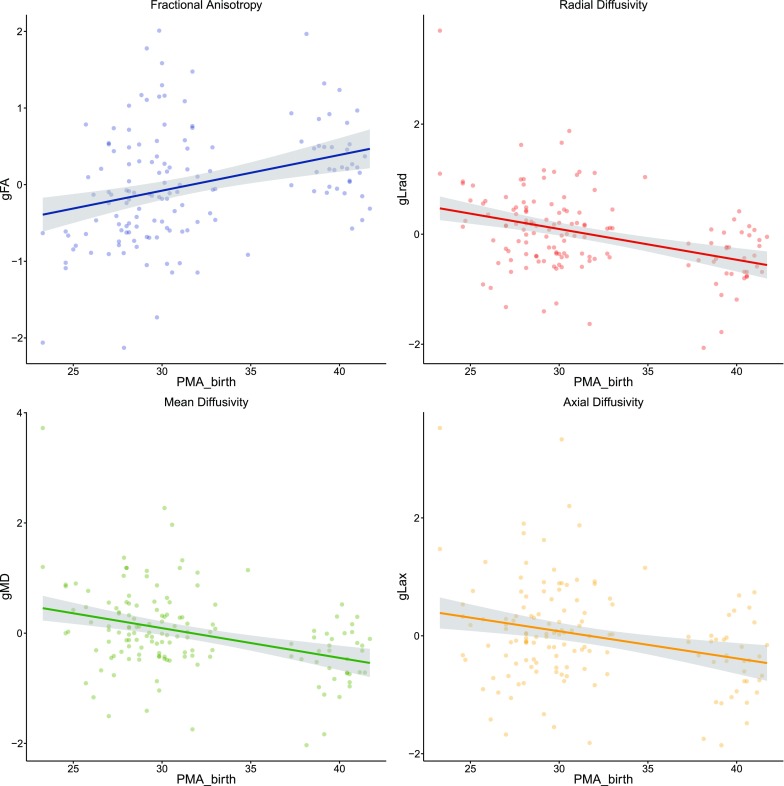
Associations between PMA birth and general measures of fractional anisotropy (*g*_FA_) mean diffusivity (*g*_MD_), radial diffusivity (*gλ*_rad_) and axial diffusivity (*gλ*_ax_). Regression lines and 95% CIs (*shaded*) are shown for linear regression models between PMA at birth and white matter microstructure, corrected for age at scan and sex

After adjustment for age at scan and sex we found significant associations between gestational age (GA) at birth and general measures of: FA (*g*_FA_), *β* 0.305 (*p* < 0.001); MD (*g*_MD_), *β* −0.351 (*p* < 0.001), *λ*_rad_ (*gλ*_rad_) *β* −0.363 (*p* < 0.001); and *λ*_ax_ (*gλ*_ax_) *β* −0.300 (*p* < 0.001) (Fig. 4). In summary, those infants born preterm exhibited less ‘mature’ microstructure (less coherent water diffusion and a greater general magnitude of water molecular diffusion) across their white matter tracts than controls. Moreover, we found a dose-dependent effect of GA at birth across all general white matter indices, such that more premature birth was associated with generally less optimal white matter microstructure.

**Fig. 5:**
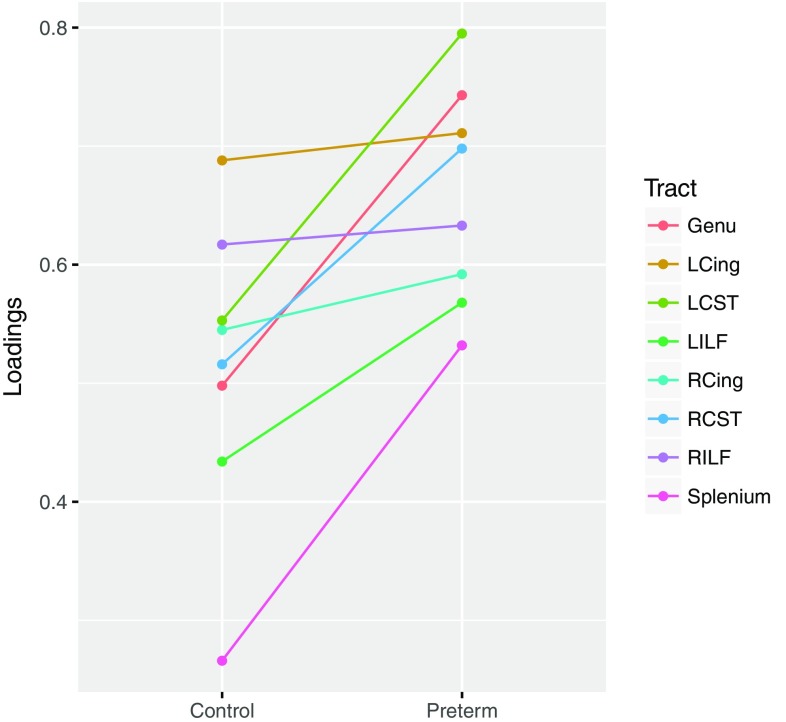
Differences in tract loadings for *g*_FA_ between control and preterm, in comparison to overall loadings

In view of variations in newborn network connectivity (van den Heuvel et al. 2015), we considered whether individual tract loading of FA might differ between preterm and term groups. In exploratory analyses we found that loadings appeared qualitatively higher in callosal and corticospinal tracts for preterm versus control infants, but there was little evidence for group difference in tract loading in association fibers (Fig. 5). Formal tests of these differences using Fisher’s *Z* broadly confirmed this pattern for genu (*z* = 2.0593, *p* = 0.0395) and left CST (*z* = 2.3185, *p* value = 0.0204), though differences were not significant in the splenium (*z* = 1.6072, *p* value = 0.1080) and right CST (*z* = 1.4674, *p* value = 0.1423). This pattern was also present for *λ*_rad_ and MD, though statistical tests indicated only trend-level or weaker differences for *λ*_rad_ (genu: *z* = 1.8146, *p* = 0.0696; splenium: *z* = 1.8551, *p* = 0.0636; left CST: *z* = 1.6845, *p* = 0.0921; and right CST *z* = 1.2033, *p* = 0.2289) with the differences in the same direction for MD being smaller and non-significant.

## Social cognitive ability and measures of general white matter microstructure

There were no significant associations between any general water diffusion parameter and a sensitive measure of emergent social cognition derived from an eye-tracking task battery at 7 months (all *β*_absolute_ ≤ 0.123, all *p* values ≥0.265), and nor were there any significant effects of group within the model (all *β*_absolute_ ≤ 0.099, all *p* values ≥0.378; Table 3). There was no relationship between water diffusion parameters and emergent social cognition in the genu (all *p* values ≥0.15) or splenium (all *p* values ≥0.064) of the corpus callosum. Social preference scores for each task (proportional looking time at social versus general image content) are shown in Supplemental Table 1.

**Table 3:** Regression models of water diffusion measures and group membership on social cognitive performance

Diffusion parameter		Group
*g*_FA_	0.076 (0.551)	0.099 (0.396)
*g*_MD_	−0.123 (0.265)	0.081 (0.478)
*gλ*_rad_	−0.116 (0.301)	0.080 (0.490)
*gλ*_ax_	−0.116 (0.295)	0.097 (0.378)

Standardized *β*s (*p* values); imaging and cognitive variables corrected for respective age at sampling prior to being entered into the models, which included gender as a covariate

*g*_*FA*_ general component of fractional anisotropy, *g*_*MD*_ general component of mean diffusivity, *gλ*_*rad*_ general component of radial diffusivity, *gλ*_*ax*_ general component of axial diffusivity

## Discussion

In the human newborn brain microstructural properties of major white matter tracts are highly correlated with one another, which allows for the extraction of a general measure for each of four common water diffusion MRI parameters. This result suggests that individual differences in white matter microstructure during development are to a substantial degree common among tracts, and not a phenomenon that primarily affects specific individual tracts. Furthermore, the nature of between tract correlations is altered by the environmental exposure of preterm birth. Since global white matter microstructure contributes to the neural foundation of higher cognitive function in later life (Deary et al. 2010), and the factor loadings show remarkable similarity to those reported in adulthood (Penke et al. 2010), the data suggest that the fundamental white matter architecture required to support cognition is established as a generalized process during gestation, and that this is vulnerable to the environmental stress of preterm birth.

Inter-tract correlations were of similar strength for FA, MD and *λ*_ax_ but were weaker for *λ*_rad_ (Table 1). In the newborn period, before myelination is widespread, FA in white matter increases in association with maturation of axonal membrane structure, and increases in axonal caliber and oligodendrocyte number. MD in white matter is high around the time of birth but decreases over the first few months of postnatal life as brain water content lowers and localized restriction of water increases due to increased cell density and other factors (Huppi et al. 1998; Neil et al. 1998; Wimberger et al. 1995; Nomura et al. 1994; Morriss et al. 1999). Our data suggest that these processes affect the major tracts similarly around term equivalent age. The observation that *λ*_ax_ was highly correlated between tracts could reflect the fact that neuronal migration has largely been completed by 24 weeks’ gestation so the axonal skeleton of major tracts is established (Bystron et al. 2008). *λ*_rad_ was relatively weakly correlated between tracts, which could be explained by variation in myelination, which is known to be tract-specific (Kinney et al. 1988).

Having established that microstructural properties of tracts are substantially shared in the newborn, we next considered whether this relationship is modified by the environmental stress of preterm birth. After controlling for age at scan and sex, we found that the latent general measures of each of the four water diffusion parameters differed between preterm and control groups (Fig. 3): *g*_FA_ was lower and *g*_MD_ higher in preterms compared with healthy infants born at term. These data are consistent with studies that have used voxel- and tractography-based approaches to study the effect of preterm birth on the developing brain (Pannek et al. 2014; Ball et al. 2010; Anblagan et al. 2015), but methodological factors have left uncertainty about the extent to which microstructural change is a local versus a generalized process. Here, we demonstrate that preterm birth is associated with generalized differences across a functionally relevant representation of network architecture. Within this, however, we also found that group differences were most marked in projection and callosal fibers, which had higher loadings than association fibers in preterm infants compared with controls. Since neonatal water diffusion parameters are biomarkers of later neurodevelopmental function after preterm birth (Counsell et al. 2008; van Kooij et al. 2012; Boardman et al. 2010), the data presented here suggest that general properties of white matter microstructure could underlie the high prevalence of impairment seen in children and adults born preterm.

We found no relationship between general properties of any of the four water diffusion parameters and measures of infant social cognition derived from eye-tracking. The cognitive measure was selected because it discriminates between typically developing children and those with atypical cognitive trajectories, including those born preterm, and has been validated for use in infancy (Young et al. 2009; Ozonoff et al. 2010; Chawarska et al. 2013; Jones and Klin 2013; Telford et al. 2016; Gillespie-Smith et al. 2016). There are plausible explanations for this. First, general white matter ‘integrity’ is most closely associated with information-processing speed in adulthood but it is less predictive of other aspects of cognition (Ritchie et al. 2015b). Second, although processing speed is considered to be a foundational competence for other cognitive abilities in adulthood this relation may not hold true in infancy (Salthouse 1996; Ritchie et al. 2015b). Thus, in the infant, social cognition may develop on an independent trajectory relative to general processing abilities or emerging intelligence (Adolphs 1999). Further study is required to determine whether *g*_WM_ relates to other aspects of infant cognition, such as sustained attention and memory. Longitudinal study will be required to determine whether foundational general measures of neonatal white matter microstructure influence later cognitive functions that are more reliant on information transfer efficiency.

Brain structure, including dMRI measures in white matter, and intelligence are all highly heritable; twin studies suggest that up to 60% of inter-individual variation in dMRI measures are attributable to genetic factors (Thompson et al. 2001; Toga and Thompson 2005; Geng et al. 2012; Shen et al. 2014). Common genetic variants and epigenetic modifications modify the risk of white matter disease associated with preterm birth (Boardman et al. 2014; Krishnan et al. 2016; Dutt et al. 2011; Sparrow et al. 2016), but to our knowledge these associations have not been tested using a more functionally tractable set of brain biomarkers. We speculate that considering general measures of network architecture alongside tract-specific measures in imaging genetic studies will be useful for understanding the genetic and epigenetic determinants of connectivity in the newborn.

A limitation of this study is that we were unable to investigate the relationship between dMRI parameters of tracts that serve social cognition in adulthood, such as the arcuate fasciculus and fornix, and infant social cognition. Although PNT can segment these tracts from adult data (Clayden et al. 2007), we could not identify them reliably in the training set of neonatal data because of lower image resolution inherent to neonatal dMRI acquisitions.

A second limitation is that we did not examine other factors that may have contributed to white matter injury in the preterm group, such as bronchopulmonary dysplasia or punctate white matter lesions, because a much larger sample would have been required to adjust for these factors (Ball et al. 2010; Bassi et al. 2011). In addition, group sizes were unequal in the secondary analysis of the effect of preterm birth on component loadings; the preterm group was larger and thus could have contributed more strongly to the principal component score, influencing group comparisons. Consequently, although we found a statistically significant group effect for FA, MD and *λ*_rad_ in the genu and CST, we cannot be certain that group differences are confined to these tracts alone. Though exploratory, these findings raise the possibility that preterm birth also subtly alters the correlational structure of infant white matter tracts with respect to specific classes of tract.

In summary, a latent general measure accounts for almost half of the variance of white matter tract microstructure in the newborn brain. Given that major white matter tracts constitute the neuroanatomical foundation of cognitive neural systems, our study indicates that a facsimile the network architecture for intelligence is established by birth, and that is it is vulnerable to early exposure to extra-uterine life.

## Electronic supplementary material

Below is the link to the electronic supplementary material.

Supplementary material 1 (DOCX 57 kb)
