# Supplementary material for: A latent measure explains substantial variance in white matter microstructure across the newborn human brain
Source: Brain Struct Funct. 2017 Jun 6;222(9):4023–33. doi: 10.1007/s00429-017-1455-6 (PMC5686254; doi:10.1007/s00429-017-1455-6)
Supplement: Supplementary file 1 — Supplementary material 1 (DOCX 57 kb) [file 429_2017_1455_MOESM1_ESM.docx]

Supplemental table 1. Social preference score for each task.

|  | Infants born preterm  (n=59) | Infants born at term  (n=24) | Mean difference | *p*-value |
| --- | --- | --- | --- | --- |
| Static face^a^ | 0.243 | 0.315 | -0.072 | 0.25 |
| Face in array of non-social images^b^ | 0.217 | 0.256 | -0.040 | 0.45 |
| Naturalistic scene^c^ | 0.437 | 0.477 | -0.040 | 0.39 |

^a^ Proportional looking time to the eye region relative to overall looking time at a static face

^b^ Proportional looking time at an image of a face within an array of non-social images

^c^ Proportional looking time at social content within a naturalistic scene relative to overall looking time at the stimulus
